# Supplementary material for: Association of Sodium, Potassium and Sodium-to-Potassium Ratio with Urine Albumin Excretion among the General Chinese Population
Source: Nutrients. 2021 Sep 29;13(10):3456. doi: 10.3390/nu13103456 (PMC8539080; doi:10.3390/nu13103456)
Supplement: Supplementary file 1 [file nutrients-13-03456-s001.zip › nutrients-1373863-supplementary.pdf]

Table S1. Odds ratio (OR) and 95% confidence interval of albuminuria at each quintile of urinary index using mixed effects logistic regression model.

**Factors**	**Model 1**			**Model 2**			**Model 3**		
Sodium (gram/day)	OR	95% CI	*P*	OR	95% CI	*P*	OR	95% CI	*P*
Q1 (≤2.83)	Ref			Ref			Ref		
Q2 (2.84~3.66)	1.284	0.878~1.878		1.328	0.906~1.946		1.303	0.878~1.933	
Q3 (3.67~4.50)	1.523	1.040~2.231	0.000	1.563	1.064~2.294	0.000	1.420	0.955~2.111	0.000
Q4 (4.51~5.65)	2.150	1.482~3.121		2.228	1.528~3.248		2.139	1.446~3.164	
Q5 (>5.65)	2.536	1.731~3.715		2.632	1.784~3.882		2.154	1.431~3.242	
Potassium (gram/day)									
Q1 (≤1.09)	Ref			Ref			Ref		
Q2 (1.10~1.35)	0.948	0.680~1.323		0.937	0.671~1.309		0.919	0.649~1.302	
Q3 (1.36~1.61)	0.662	0.463~0.947	0.073	0.667	0.465~0.956	0.079	0.681	0.468~0.990	0.239
Q4 (1.62~1.99)	0.759	0.534~1.078		0.763	0.535~1.087		0.779	0.538~1.128	
Q5 (>1.99)	0.674	0.468~0.972		0.661	0.457~0.954		0.719	0.488~1.059	
Na/K ratio									
Q1 (≤3.33)	Ref			Ref			Ref		
Q2 (3.34~4.22)	1.174	0.806~1.709		1.204	0.825~1.756		1.136	0.769~1.679	
Q3 (4.23~5.15)	1.243	0.857~1.803	0.000	1.269	0.872~1.846	0.000	1.185	0.804~1.748	0.001
Q4 (5.16~6.46)	1.640	1.151~2.336		1.659	1.160~2.371		1.613	1.113~2.338	
Q5 (>6.46)	2.251	1.602~3.164		2.332	1.649~3.297		1.953	1.356~2.813	

Notes: Q indicates quintile. Model 1 examined the association of sodium and potassium or Na/K ratio quintiles with the odds of albuminuria without adjustment. Model 2 adjusted age and sex; model 3 adjust age, sex, region (north/south), smoking, drinking, physical activity, BMI, diabetes, systolic blood pressure and antihypertensive medication use.
